# Return to Sport After Arthroscopic Bankart Repair in Professional Male Football Players

**DOI:** 10.3390/jcm15135259

**Published:** 2026-07-06

**Authors:** Yener İnce, Muhammed Yusuf Afacan, Goker Utku Deger, Tolgahan Korkmaz

**Affiliations:** 1 Department of Orthopaedics and Traumatology, Acibadem Maslak Hospital, Istanbul 34457, Turkey; yenerince@yahoo.com; 2 Department of Orthopaedics and Traumatology, Istanbul Physical Therapy and Rehabilitation Training and Research Hospital, Istanbul 34180, Turkey; drmyaorto@gmail.com; 3 Department of Orthopaedics and Traumatology, Acibadem Taksim Hospital, Istanbul 34373, Turkey; gokerdeger@hotmail.com; 4 Department of Orthopaedics and Traumatology, Başaksehir Cam and Sakura City Hospital, Istanbul 34480, Turkey

**Keywords:** shoulder dislocation, arthroscopy, return to sport, soccer, joint instability, athletic injuries

## Abstract

**Background/Objectives**: Traumatic anterior shoulder instability is a clinically relevant problem in professional football (soccer) players, for whom restoration of shoulder function, postoperative stability and timely return to sport are critical. Despite a growing body of evidence supporting arthroscopic Bankart repair in athletic populations, data on sport-specific outcomes in professional male football players remain limited. **Methods**: Twenty-two professional male football (soccer) players who underwent arthroscopic Bankart repair following first-time anterior traumatic shoulder instability between 2011 and 2021 were retrospectively analyzed. No control group was included. The Rowe score and American Shoulder and Elbow Surgeons (ASES) score were assessed preoperatively and at 12 months postoperatively. Return-to-sport time was defined as the time from surgery to return to competitive match play and was recorded in weeks. Recurrent instability was assessed throughout the follow-up period. IBM SPSS Statistics, v27 was used for statistical analyses. **Results**: The median age was 25 years old (15–34). The right shoulder was involved in 15 patients (68%) and the left in 7 (32%). The median time to return to sport was 18 weeks (range, 14–25). During a median follow-up of 6.5 years (range, 4–14), recurrent instability occurred in one patient (4.5%) at 14 months after surgery. After surgery, both functional scores improved significantly. The median preoperative Rowe score increased from 55 (range, 35–60) to 100 (range, 85–100) (*p* < 0.001), and the median ASES score improved from 55 (range, 40–68) to 100 (range, 85–100) (*p* < 0.001) at 12 months. Postoperative ASES and Rowe scores were positively correlated (r = 0.666, *p* = 0.001). Neither score was significantly associated with return-to-sport timing. **Conclusions**: In this retrospective series of professional male football players with first-time anterior shoulder instability, arthroscopic Bankart repair was associated with significant improvements in 12-month Rowe and ASES scores, a median return-to-sport time of 18 weeks, and a low observed recurrence rate during long-term follow-up. These findings should be interpreted within the context of the study’s retrospective design and limited sample size.

## 1. Introduction

Traumatic anterior shoulder instability is a clinically important problem in athletes, as it may compromise shoulder function, affect sport participation and challenge return to competition [1,2,3,4]. The risk of recurrent instability after nonoperative management is particularly high in young male athletes participating in contact and collision sports [5]. Arthroscopic Bankart repair is widely used for appropriately selected patients and has demonstrated satisfactory clinical outcomes with reported return-to-sport rates exceeding 90% [2,6,7] In parallel, contemporary sports medicine literature increasingly evaluates postoperative success not only in terms of recurrent instability but also with respect to functional recovery and return to sport [8,9].

Professional football (soccer) exposes athletes to frequent tackles, falls, aerial challenges, and player-to-player contact. Recent reports in professional male football players have shown that shoulder dislocation remains a clinically relevant injury and affects player availability and performance outcomes [10,11]. As the world’s most popular sport and one of the largest global sports industries, professional football places considerable emphasis on player availability and rapid return to preinjury performance. Consequently, injuries affecting shoulder stability may have substantial sporting and economic consequences for both players and clubs. Sport-specific clinical studies have shown that arthroscopic Bankart repair can provide favorable functional outcomes and support a return to sport in soccer players, handball players and football players [12,13,14,15]. Although recurrence rates after arthroscopic shoulder stabilization appear to be lower in soccer than in several other contact and collision sports, such as ice hockey, wrestling, and rugby, shoulder instability remains a clinically important problem in professional football because even a single episode may result in prolonged absence from competition [16].

Most previous studies have evaluated heterogeneous athletic populations, included different surgical procedures, or focused on upper-extremity-dominant sports [5,8,17]. Despite the growing body of evidence, data focused on isolated arthroscopic Bankart repair in professional football players remain limited [11,12,15]. In addition, data regarding return-to-sport timing, long-term recurrent instability, and functional outcomes in homogeneous cohorts of professional football players remain scarce. Systematic reviews have also shown that outcomes of return-to-sport after anterior shoulder instability surgery vary according to procedure type, athletic level, and follow-up time, underscoring the need for further sport-specific evidence [6,7,8,9].

Therefore, the mid- to long-term outcomes of isolated arthroscopic Bankart repair in professional male football players are insufficiently characterized. Accordingly, the present study aimed to evaluate return-to-sport time, functional outcomes, and recurrent instability after arthroscopic Bankart repair in professional male football players with traumatic anterior shoulder instability.

## 2. Materials and Methods

This retrospective study evaluated return to sport, functional outcomes, and recurrent instability after arthroscopic Bankart repair in professional male football (soccer) players with traumatic anterior shoulder instability. Medical Research Ethics Committee (ATADEK, decision no. 2026-05/221) approval was obtained. All patients were informed and gave written consent before inclusion.

Twenty-two professional male football players were included. A professional football player was defined as an athlete registered with a club competing in the Turkish professional football league (first, second and third leagues). Patients were retrospectively identified from the institutional surgical database over the study period from 2011 to 2021. No control group was included. Eligibility criteria were as follows: (1) traumatic first-time anterior shoulder dislocation, (2) professional football participation, (3) arthroscopic Bankart repair for an anterior capsulolabral lesion, including anterior labral periosteal sleeve avulsion lesions when present, without the need for additional intra-articular procedures, and (4) availability of complete preoperative and postoperative clinical records with adequate follow-up. Goalkeepers were excluded due to different shoulder loading patterns and injury mechanisms. Preoperative CT and MRI images, together with intraoperative arthroscopic findings, were retrospectively reviewed. CT images were used for the assessment of fractures, glenoid bone loss and Hill-Sachs lesions. Patients with Bankart lesions, including anterior labral periosteal sleeve avulsion (ALPSA) lesions, were included. Patients with bony Bankart lesions or glenoid bone loss, as assessed on en face 3D CT images, were excluded. For the evaluation of Hill-Sachs lesions preoperative MRI and arthroscopic findings were used in addition to CT. Patients with engaging Hill-Sachs lesions requiring remplissage were excluded. Previous shoulder dislocation, fracture-dislocation, previous surgery on the affected shoulder, or any additional procedure involving the humeral head were excluded.

**Figure 1:**
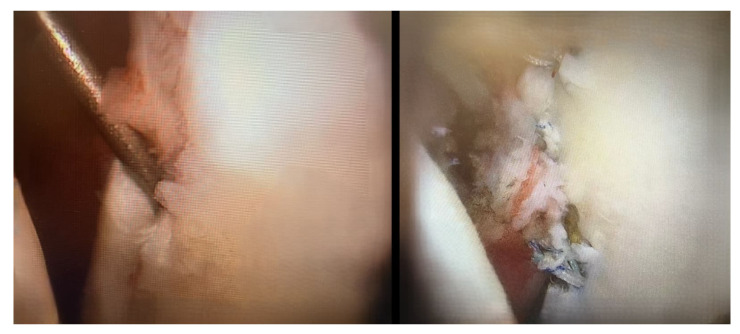
Arthroscopic view of the anteroinferior glenoid labrum before and after Bankart repair.

Patients underwent surgical stabilization approximately 4 weeks after the initial dislocation. All procedures were performed arthroscopically by the same experienced orthopedic shoulder surgeon using a standardized operative technique. Under general anesthesia, patients were positioned in the beach-chair position. After standard sterile preparation and draping, posterior, anterior, and anterosuperior arthroscopic portals were established. Diagnostic arthroscopy was routinely performed in every case to confirm the anterior capsulolabral lesion and assess associated intra-articular pathology, including the glenoid labrum, capsule, glenohumeral ligaments and rotator cuff. The detached anterior labrum was mobilized with a periosteal elevator to allow adequate capsulolabral shift and anatomic reduction. Arthroscopic Bankart repair was then performed using bioabsorbable polyetheretherketone (PEEK) suture anchors (GRYPHON; DePuy Synthes, Johnson & Johnson MedTech, Raynham, MA, USA) to restore the anteroinferior labrum and capsulolabral complex (Figure 1). Three or four anchors were used according to lesion morphology.

Postoperatively, the shoulder was immobilized in a sling for 3 weeks. All patients followed the same rehabilitation protocol under the supervision of the treating team. During the first 3 weeks, external rotation was restricted, and passive and assisted active range-of-motion exercises were initiated. Active exercises were started at 6 weeks postoperatively. Strengthening and endurance exercises were introduced at 3 months. Progressive sport-specific loading was subsequently permitted, and return-to-play was allowed after completion of the rehabilitation program based on clinical recovery, restoration of shoulder function and the absence of instability symptoms. Patients were evaluated weekly during the first 3 postoperative weeks, every 3 months during the first postoperative year, and annually thereafter. Clinical assessment included the Rowe score and the American Shoulder and Elbow Surgeons (ASES) score, both recorded preoperatively and at 12 months postoperatively. Return to sport was defined as the time from surgery to return to competitive match play and was recorded in weeks. Return-to-play readiness was determined by the treating surgeon and rehabilitation team based on clinical criteria, including restoration of full pain-free range of motion, symmetric shoulder strength, absence of apprehension, and sport-specific functional progression without instability symptoms. No formal validated return-to-sport test was applied. Recurrent instability was defined as a postoperative glenohumeral dislocation and was recorded until the last follow-up.

IBM SPSS, version 27 (IBM Corp., Armonk, NY, USA) was used for statistical analyses. Continuous and categorical variables were presented as median (range) and number (percentage), respectively. The Shapiro–Wilk test was used for normality assessment of continuous variables. Nonparametric methods were used for not-normally distributed variables. The Wilcoxon signed-rank test was used for the comparison of preoperative and postoperative functional outcome scores. Spearman rank correlation coefficients were used for correlations between postoperative functional scores and return-to-sport time. Statistical significance was defined as *p* value of <0.05.

## 3. Results

A total of 22 professional male football players were included. The median age at surgery was 25 years (range, 15–34 years), and the median follow-up was 6.5 years (range, 4–14 years). The right shoulder was involved in 15 patients (68.2%) and the left shoulder in seven patients (31.8%). The median number of anchors used during surgery was 3 (range, 3–4). Nineteen players (86.4%) were right-hand dominant, and three (13.6%) were left-hand dominant. Six players (27.3%) competed in the Turkish First League, nine (40.9%) in the Second League, and seven (31.8%) in the Third League. According to playing position, seven players (31.8%) were defenders, ten (45.5%) were midfielders, and five (22.7%) were forwards. The median time to return to sport was 18 weeks (range, 14–25 weeks). During follow-up, recurrent instability was observed in one patient (4.5%) at 14 months, whereas 21 patients (95.5%) remained stable during follow-up (Table 1).

Significant postoperative improvements were observed in both functional outcome measures. The median Rowe score increased from 55 (range, 35–60) preoperatively to 100 (range, 85–100) at 12 months, with a median improvement of 45 points and a large effect size (*p* < 0.001; effect size r = 0.88). Similarly, the median ASES score improved from 55 (range, 40–68) preoperatively to 100 (range, 85–100) postoperatively, with a median improvement of 45 points and a large effect size (*p* < 0.001; effect size r = 0.88) (Table 2).

Spearman correlation analysis demonstrated a significant positive correlation between postoperative ASES and Rowe scores (r = 0.666, *p* = 0.001). No significant associations were found between functional outcome scores and return-to-sport timing (postoperative ASES: r = −0.369, *p* = 0.091; postoperative Rowe: r = 0.107, *p* = 0.635) (Table 3).

## 4. Discussion

In a selected group of professional male football players with traumatic first-time anterior shoulder instability, arthroscopic Bankart repair was associated with significant improvement in Rowe and ASES scores at 12 months, return to sport at a median of 18 weeks, and a low recurrence rate (4.5%) at long-term follow-up.

The low recurrence rate and favorable return-to-sport profile observed in this study are consistent with previous sport-specific reports demonstrating that arthroscopic Bankart repair can restore postoperative stability and permit return to competitive play in high-demand athletes [12,13,14,15,18]. Pasqualini et al. reported significant functional improvement, a low recurrence rate and satisfying return to sport following arthroscopic Bankart repair, providing the most directly comparable football-specific evidence to the present findings [12]. Similarly, Saper et al. demonstrated successful postoperative stability and return to sport in American football players, whereas Pavlik et al. and Perret et al. reported favorable postoperative outcomes in professional handball and Australian rules football players, respectively [13,14,15]. A more recent study in professional martial arts athletes also showed that arthroscopic Bankart repair can provide durable functional improvement and reliable return to sport in another high-demand sporting population [18]. More broadly, recent football- and contact-athlete-focused reviews have continued to support arthroscopic soft-tissue stabilization in carefully selected athletes without substantial bone loss or other indications for augmentation or bony reconstruction [1,2,3,4,5,6]. Epidemiologic data from professional male football further underscore the clinical relevance of shoulder instability in this population and its potential effect on player availability and subsequent performance [10,11].

The substantial postoperative improvement in both Rowe and ASES scores at 12 months after surgery is also in line with the available literature on athletes undergoing arthroscopic Bankart repair. Significant postoperative improvement in functional scores has been reported in soccer players, professional handball players, and other athletic cohorts treated with arthroscopic stabilization [12,13,17,18]. In addition, systematic reviews have concluded that arthroscopic Bankart repair generally provides favorable clinical and functional outcomes for anterior shoulder instability when patient selection is appropriate and major bone loss is absent [2,6]. From a clinical standpoint, this issue is especially important in football players because postoperative success in this population requires restoration of shoulder function that is sufficient not only for daily activity, but also for repetitive training exposure, contact situations and sustained high-level competition [3,8,10,11]. Although no significant correlations were identified between functional outcome scores and return-to-sport timing in the present study, this finding should be interpreted with caution, as the small sample size (*n* = 22) may have limited the statistical power to detect existing associations. Furthermore, both postoperative Rowe and ASES scores demonstrated a notable ceiling effect, with most athletes achieving near-maximal values. This restricted score range may have further reduced the ability to detect correlations with return-to-sport timing and should be considered when interpreting the correlation analyses. Therefore, the correlation analyses should be interpreted cautiously. It should be noted that functional outcomes were assessed at the routine 12-month postoperative evaluation, whereas recurrent instability was assessed throughout the entire follow-up period (median, 6.5 years). Accordingly, the present study provides mid-term functional outcome data together with long-term recurrence data. Although this approach reflects routine clinical follow-up in our institution, long-term functional outcomes beyond 12 months remain to be established in future studies.

The findings should also be interpreted considering the indication and timing of surgery. All patients in the present study underwent arthroscopic stabilization after a first-time traumatic dislocation and relatively early after the index event. Randomized trials have shown that early arthroscopic stabilization after a first anterior shoulder dislocation can reduce recurrent instability compared with nonoperative treatment in young active patients [19,20]. In addition, comparative data in athletes have suggested that the surgical strategy after first-time instability should be individualized according to risk profile and structural pathology, rather than determined solely by athletic status [1,2,4].

Arthroscopic Bankart repair remains the most frequently performed stabilization procedure for anterior shoulder instability, although the use of augmentation procedures has increased in selected patients with greater structural bone loss [5,7]. Contemporary evidence suggests that remplissage may improve outcomes compared with isolated Bankart repair and provide results comparable to the Latarjet procedure in appropriately selected patients, whereas the Latarjet procedure is generally reserved for patients with critical glenoid bone loss. In the present study, the low long-term recurrence rate is consistent with previous evidence supporting early stabilization in carefully selected athletes with soft-tissue lesions amenable to Bankart repair. However, because this was a retrospective study without a nonoperative or surgical control group, these findings should be interpreted as demonstrating favorable outcomes in a selected cohort rather than evidence of superiority over nonoperative treatment, the Latarjet procedure, remplissage, or other stabilization strategies.

The correlation analysis provides an additional clinically relevant perspective. The significant positive association between postoperative ASES and Rowe scores suggests agreement between two commonly used measures of postoperative shoulder recovery, which is concordant with prior athletic stabilization studies that have used similar outcome instruments to characterize recovery after arthroscopic Bankart repair [13,17,18,21]. In contrast, no significant relationship was seen between postoperative functional scores and time to return to sport. This finding is not unexpected because return to sport after anterior shoulder stabilization is recognized as a multifactorial endpoint influenced by symptom resolution, recurrence risk, psychological readiness, rehabilitation progress, medical clearance and sport-specific demands [8,9,21,22,23,24,25,26]. Criteria-based return-to-sport frameworks, rehabilitation-based progression models, and composite readiness tests have all been proposed to improve decision-making after shoulder stabilization, further underscoring that patient-reported shoulder function alone does not fully determine the timing of return to competition [8,21,23,24]. Moreover, systematic reviews have shown substantial variability in return-to-sport outcomes according to sport type, surgical procedure, athletic level and duration of follow-up, including in collision athletes and overhead athletes [8,9,22,25,26]. Therefore, the absence of a significant correlation between postoperative functional scores and return-to-sport timing should be interpreted cautiously. In addition, the small sample size of the present study may limit the statistical power to detect clinically meaningful associations between functional outcome measures and return-to-sport timing. Future studies with larger cohorts and objective return-to-performance variables are needed to better clarify the relationship between postoperative shoulder function and sport-specific recovery after arthroscopic Bankart repair.

This study has several strengths. First, it focused on a highly specific and clinically relevant population—professional male football players—representing a uniquely high-demand group in whom shoulder stability, functional recovery, and timely return to sport are of particular importance. Second, all patients were treated with the same arthroscopic technique using a standardized surgical approach and postoperative rehabilitation protocol, which likely reduced treatment-related heterogeneity. Third, the study provided long-term follow-up and evaluated not only recurrent instability and functional outcome scores but also return-to-sport timing, which is a particularly meaningful endpoint in athletic populations.

The most important limitations of this study are the retrospective design and a relatively small sample size. The study also lacked a control group, which precludes generalizability and definitive conclusions regarding the superiority of arthroscopic Bankart repair over other treatment strategies. Although return to sport was recorded, it was defined solely as return to competitive match play. Return to training and return to preinjury performance level were not assessed separately, and no standardized objective criteria for return to sport were available. Data regarding return to preinjury competitive level, playing time, match exposure and objective performance metrics were not available. Furthermore, functional outcomes were available only at the routine 12-month follow-up, whereas recurrent instability was assessed over long-term follow-up; therefore, long-term functional recovery could not be evaluated. The group consisted exclusively of professional male football players; therefore, the findings may not be directly applicable to female athletes, recreational athletes or other sporting populations. Other factors that may influence postoperative outcomes—such as subtle differences in capsular tissue quality, lesion extent, psychological readiness, and sport-specific exposure—could not be fully controlled in this retrospective design.

## 5. Conclusions

In this selected series of professional male football players without major bone loss or additional procedures, arthroscopic Bankart repair was associated with improved 12-month Rowe and ASES scores, a median return to sport at 18 weeks, and a low observed recurrence rate in long-term follow-up. These findings should be interpreted in light of the retrospective study design and the selected patient population.

## Figures and Tables

**Table 1:** Baseline demographic, clinical, and surgical characteristics of patients.

Variable	Patient, *n* = 22
Age, years	25 (15–34)
Male sex, ***n*** (%)	22 (100)
Dominant arm, ***n*** (%)	
-Right	19 (86.4)
-Left	3 (13.6)
League level, ***n*** (%)	
-First league	6 (27.3)
-Second league	9 (40.9)
-Third league	7 (31.8)
Playing position, ***n*** (%)	
-Defender	7 (31.8)
-Midfielder	10 (45.5)
-Forward	5 (22.7)
Operated shoulder, ***n*** (%)	
-Right	15 (68.2)
-Left	7 (31.8)
Follow-up duration, years	6.5 (4–14)
Time to return to sport, weeks	18 (14–25)
Recurrent instability, ***n*** (%)	1 (4.5)

**Table 2:** Comparison of preoperative and postoperative functional outcomes.

Functional Outcome	Preoperative	Postoperative	*p* Value	Effect Size (r)
Rowe score	55 (35–60)	100 (85–100)	<0.001	0.88
ASES score	55 (40–68)	100 (85–100)	<0.001	0.88

**Table 3:** Correlation analysis.

Variables	Correlation Coefficient (r)	*p* Value
Postoperative ASES vs. postoperative Rowe	0.666	0.001
Postoperative ASES vs. return-to-sport time	–0.369	0.091
Postoperative Rowe vs. return-to-sport time	0.107	0.635

## Data Availability

Data supporting reported results can be shared upon request.

## Abbreviations

The following abbreviations are used in this manuscript:

ASES	American Shoulder and Elbow Surgeons
CT	Computed Tomography
MRI	Magnetic Resonance Imaging
PEEK	Polyetheretherketone
SPSS	Statistical Package for the Social Sciences

## References

[1] Brinkman J.C., Damitio E., Tokish J.M. (2024). Arthroscopic Management of the Contact Athlete with Anterior Instability. Clin. Sports Med..

[2] Hossein Zadeh R., Daliri M., Sadeghi M., Hossein Zadeh R., Sahebi M., Moradi A., Samei M., Ebrahimzadeh M.H. (2024). Arthroscopic Bankart repair vs. Latarjet procedure for recurrent shoulder instability: A meta-analysis of clinical outcomes and complication rates in general and athletic populations. J. Shoulder Elb. Surg..

[3] Lanham N.S., Davidson I., Graefe B., Updegrove G. (2025). Evaluation and Management Anterior Shoulder Instability Among Football Players. Curr. Rev. Musculoskelet. Med..

[4] Meyer A.M., Lorentz S.G., Droz L.G., Ralph J.E., Lau B.C. (2025). Management of anterior shoulder instability in the contact athlete: A narrative review. Ann. Jt..

[5] Lau E.N., Brown C.L., Saggar R., Mullen J., Olawin A., Lunn K., Lin A., Hicks J.J. (2026). Managing Shoulder Instability in the Contact and Collision Athlete. Curr. Rev. Musculoskelet. Med..

[6] Asiri F.A.M., Alqhtani A.A., Assiri A.H., Alqahtani M.H., Tedla J.S., Awwadh B.A.A. (2024). Systematic Review of Arthroscopic Bankart Repair Outcomes for Anterior Shoulder Instability. Med. Sci. Monit..

[7] Ahmed A.S., Gabig A.M., Dawes A., Gottschalk M.B., Lamplot J.D., Wagner E.R. (2023). Trends and projections in surgical stabilization of glenohumeral instability in the United States from 2009 to 2030: Rise of the Latarjet procedure and fall of open Bankart repair. J. Shoulder Elb. Surg..

[8] Fails A., Popchak A. (2025). Return to Competitive Sport After Anterior Shoulder Stabilization: A Scoping Review of Current Outcomes and Clearance Decision-Making Criteria. Int. J. Sports Phys. Ther..

[9] Kim M., Haratian A., Fathi A., Kim D.R., Patel N., Bolia I.K., Hasan L.K., Petrigliano F.A., Weber A.E. (2023). Can We Identify Why Athletes Fail to Return to Sports After Arthroscopic Bankart Repair? A Systematic Review and Meta-analysis. Am. J. Sports Med..

[10] Degener B.J., Theil C., Gosheger G., May P., Seidel J., Schachtrup T., Zafeiris T., Schneider K.N. (2025). Shoulder dislocations in professional male football (soccer) do not negatively affect long-term quantitative and qualitative performance parameters: A retrospective analysis of 30 in-match injuries of the German Bundesliga. J. Exp. Orthop..

[11] Schneider K.N., Zafeiris T., Gosheger G., Klingebiel S., Rickert C., Schachtrup T., Theil C. (2024). Shoulder dislocations in professional male football (soccer): A retrospective epidemiological analysis of the German Bundesliga from season 2012/2013 until 2022/2023. Knee Surg. Sport Traumatol. Arthrosc..

[12] Pasqualini I., Rossi L.A., Tanoira I., Ranalletta M. (2022). Return to sports, functional outcomes, and recurrences after arthroscopic Bankart repair in soccer players. Shoulder Elb..

[13] Pavlik A., Tátrai M., Tátrai A., Tállay A. (2021). Outcomes After Arthroscopic Anterior Shoulder Stabilization in Professional Handball Players. Orthop. J. Sport Med..

[14] Perret M., Warby S., Brais G., Hinse S., Hoy S., Hoy G. (2021). Return to Professional Australian Rules Football After Surgery for Traumatic Anterior Shoulder Instability. Am. J. Sports Med..

[15] Saper M.G., Courson J., Milchteim C., Plummer H., Andrews J.R., Ostrander R.V. (2022). Successful Outcomes and Return to Sport After Arthroscopic Bankart Repair in National Collegiate Athletic Association and National Football League Football Players. Clin. J. Sport Med..

[16] Hohmann E. (2024). Editorial Commentary: Recurrence Rates Following Arthroscopic Bankart Repair Differ Among Contact and Collision Sports and Are Higher in Collision Sports. Arthroscopy.

[17] Hurley E.T., Davey M.S., Mojica E.S., Fried J.W., Gaafar M., Pauzenberger L., Mullett H. (2022). Evaluation of factors associated with successful 5-year outcomes following arthroscopic Bankart repair in athletes. Knee Surg. Sport Traumatol. Arthrosc..

[18] İnce Y., Korkmaz T. (2026). Arthroscopic Bankart Repair for the Management of Anterior Shoulder Instability in Professional Martial Arts Athletes: 5-Year Follow-up Results. Orthop. J. Sport Med..

[19] Minkus M., Königshausen M., Pauly S., Maier D., Mauch F., Stein T., Greiner S., Moursy M., Scheibel M. (2021). Immobilization in External Rotation and Abduction Versus Arthroscopic Stabilization After First-Time Anterior Shoulder Dislocation: A Multicenter Randomized Controlled Trial. Am. J. Sports Med..

[20] Pougès C., Hardy A., Vervoort T., Amouyel T., Duriez P., Lalanne C., Szymanski C., Deken V., Chantelot C., Upex P. (2021). Arthroscopic Bankart Repair Versus Immobilization for First Episode of Anterior Shoulder Dislocation Before the Age of 25: A Randomized Controlled Trial. Am. J. Sports Med..

[21] Drummond Junior M., Popchak A., Wilson K., Kane G., Lin A. (2021). Criteria-based return-to-sport testing is associated with lower recurrence rates following arthroscopic Bankart repair. J. Shoulder Elb. Surg..

[22] Lin A., Barrow A.E., Charles S., Shannon M., Fox M.A., Herman Z.J., Greiner J.J., Hughes J.D., Denard P.J., Narbona P. (2023). Remplissage reduces recurrent instability in high-risk patients with on-track Hill-Sachs lesions. J. Shoulder Elb. Surg..

[23] Juré D., Blache Y., Degot M., Vigne G., Nové-Josserand L., Godenèche A., Collotte P., Franger G., Borel F., Rogowski I. (2022). The S-STARTS Test: Validation of a Composite Test for the Assessment of Readiness to Return to Sport After Shoulder Stabilization Surgery. Sport Health A Multidiscip. Approach.

[24] Kelley T.D., Clegg S., Rodenhouse P., Hinz J., Busconi B.D. (2022). Functional Rehabilitation and Return to Play After Arthroscopic Surgical Stabilization for Anterior Shoulder Instability. Sport Health A Multidiscip. Approach.

[25] Pasqualini I., Turan O.A., Hurley E.T., Frangiamore S.J., Levin J.M., Dickens J.F., Klifto C.S., Rossi L.A. (2025). Return to sports following arthroscopic Bankart repair in collision athletes: A systematic review. Shoulder Elb..

[26] Valk J., Deshpande V., Hitchens H., Zediker C., Simpson E., Parvaresh K., Kassam H. (2025). Ranges of Return to Sport Outcomes Following Anterior Shoulder Instability Surgery Are Influenced by Procedure, Athletic Level, and Follow-Up Duration: A Systematic Review. Arthrosc. J. Arthrosc. Relat. Surg..

